# Camphene as a Protective Agent in Myocardial Ischemia/Reperfusion Injury

**DOI:** 10.3390/antiox13040405

**Published:** 2024-03-28

**Authors:** Rodopi Stamatiou, Maria Anagnostopoulou, Konstantina Ioannidou-Kabouri, Chrysa Rapti, Antigone Lazou

**Affiliations:** Laboratory of Animal Physiology, School of Biology, Aristotle University of Thessaloniki, 54124 Thessaloniki, Greece; rstamatiou@uth.gr (R.S.); kioannid@bio.auth.gr (K.I.-K.); chrysarapti@gmail.com (C.R.)

**Keywords:** camphene, ischemia/reperfusion injury, oxidative stress, antioxidant activity, redox homeostasis, ferroptosis, Nrf2 signaling

## Abstract

Myocardial ischemia/reperfusion injury (I/R) and the resulting heart failure is one of the main causes of mortality and morbidity worldwide. Camphene has been shown to have anti-inflammatory and hypolipidemic properties; however, its role in the protection of the heart from ischemia and reperfusion has not been investigated. The cardioprotective role of camphene and the mechanism that mediates its action against I/R injury was evaluated in the present study. A single dose of camphene was administered in adult rats prior to ex vivo I/R induction. Infarct size was measured using 2,3,5-triphenyltetrazolium chloride (TTC) staining and cardiomyocyte injury was assessed by determining the release of the enzyme lactate dehydrogenase (LDH). Camphene pretreatment provided significant protection reducing myocardial infarct size and cell death after I/R. The effect was correlated with the reduction in oxidative stress as evidenced by the determination of protein carbonylation, GSH/GSSG ratio, the increase in mitochondrial content as determined by CS activity, and the modulation of antioxidant defense mechanisms (expression of Nrf2 and target genes and activities of CAT, MnSOD, and GR). Furthermore, ferroptosis was decreased, as demonstrated by downregulation of GPx4 expression and reduction in lipid peroxidation. The results suggest that camphene can protect the heart against I/R injury by maintaining redox homeostasis and can hold therapeutic potential for mitigating the detrimental effects of I/R in the heart.

## 1. Introduction

Acute myocardial infarction, and the ensuing heart failure, is a major cause of morbidity and mortality worldwide. Timely myocardial reperfusion, which remains the only treatment of choice up to date, can paradoxically exacerbate myocardial injury and cardiomyocyte death, known as ischemia/reperfusion (I/R) injury [1]. I/R injury is a complex process during which physiological mechanisms in cardiomyocytes are unable to maintain homeostasis. The mechanism of I/R injury is multifactorial and involves the contribution of divergent pathways, with reperfusion-induced oxidative stress being one of the main players [2]. Rapid restoration of oxygen at the onset of reperfusion overwhelms the mitochondria, leading to redox balance deregulation and massive reactive oxygen species (ROS) production, lipid peroxidation, and formation of toxic products that react with and inactivate subcellular components and further aggravate oxidative damage and overall cardiomyocyte dysfunction [1,3,4]. Mitochondrial dysfunction compromises ATP production and the ability of the cardiac cell to remain viable leading to lethal injury. Several forms of programmed cell death have been associated with I/R injury including apoptosis, necroptosis, and ferroptosis [5]. The intrinsic defense mechanisms in place, in cardiac myocytes as in most cell types, to counteract oxidative stress and diminish the buildup of toxic radicals consist of enzymatic antioxidants such as heme oxygenase-1 (HO-1), superoxide dismutase (SOD), catalase (CAT), glutathione peroxidases (GPxs) and thioredoxin (Trx), as well as the non-enzymatic antioxidants including glutathione (GSH). GSH may directly react with different free radicals, thus being the first cellular defense line against oxygen-reactive species or acting as a co-factor of antioxidant enzymes [6,7,8]. Nrf2 is a key transcriptional factor that induces antioxidant gene expression, such as HO-1, SOD, and UCP3 [9,10], and plays an important role in redox homeostasis in cardiomyocytes.

Pharmacological approaches that seek to ameliorate the effect of oxidative stress either enhancing antioxidant mechanisms or reducing ROS production have shown some promising results and are considered a potentially useful strategy in the management of I/R injury [2,11,12,13]. In addition to exogenous antioxidants, therapies targeting endogenous antioxidant systems, i.e., indirect antioxidants, have become of particular interest in the treatment or prevention of cardiovascular diseases. Endogenous antioxidant systems in the heart and/or in circulation could be activated by various exogenous molecules, and some of them have shown cardioprotective potential in clinical settings as well. In particular, the use of compounds found in everyday natural products and food as potential cardioprotective factors is of great interest [7,14].

In the present study, we used camphene, a monoterpene that can be found in various plants such as carrots, pepper, dill, fennel, nutmeg, thyme, and cannabis. Camphene is used as an additive in foods and fragrances, and it is included in essential oils as well [15]. It has been recognized to have antibiotic anti-fungal [16,17,18], anti-inflammatory, and analgesic properties [19,20], while it is also used as an expectorant [21] and antinociceptive [22]. Furthermore, camphene has been reported to exhibit a hypolipidemic action [23] while it increases the expression of apolipoprotein ApoA1 protein levels in a dose-dependent manner [24], resulting in increased formation of high-density lipoprotein (HDL) molecules [23]. Although camphene has been shown to have antioxidant properties in vitro [25], data on the antioxidant effect of camphene in animal or cell systems are very limited. A recent report demonstrated the reduction in the starvation-induced ROS generation in L6 skeletal muscle cells in the presence of camphene [26]; however, the mechanisms underlying this effect are unclear.

The aim of the current research is to characterize the potential cardioprotective effect of camphene administration in terms of infarct size reduction after myocardial I/R and to investigate the underlying mechanism of action.

## 2. Materials and Methods

### 2.1. Animals

Adult (age 2–3 months old) Wistar rats were maintained in plexiglass cages in a controlled environment, namely temperature 22 ± 1 °C, humidity 55 ± 2%, with a 12 h day/night circle, in a quiet and well-ventilated animal house. A total of 2–4 animals were housed in each cage, with free access to food and water. All animals were acclimated to the researchers who handled them and/or were involved in the euthanasia. All protocols applied were in accordance with the ethical regulations for the use of laboratory animals of the Aristotle University of Thessaloniki (13/16588), the Greek legislation on laboratory animals (“*Guidelines for the care and use of laboratory animals*”, published by the Greek government 160/1991), and the regulations of the European Union (86/609).

### 2.2. Administration of Camphene and Experimental Procedures for I/R in Isolated Hearts

Animals were randomly assigned to three groups. The control group and I/R group animals were given an intraperitoneal (i.p.) bolus of 1 mL of Tween 80 (10%) while I/R-camphene group animals were administered a single dose, 30 μg/g of body weight of camphene (Sigma Aldrich, Darmstadt, Germany) dissolved in Tween 80 (10%), 24 h before any further intervention. The dosage for camphene was based on previously published studies [27].

Animals were anesthetized with i.p. administration of ketamine/xylazine at 100 mg/kg and 10 mg/kg, respectively, (Merck, KGaA, Darmstadt, Germany) and they were injected with heparin (300 IU/kg) through the femoral vein to prevent blood coagulation and the formation of clots in the cavities of the heart. Hearts were excised and placed immediately in ice-cold Krebs–Heinseleit buffer (KHB) (25 mM NaHCO_3_, 118.5 mM NaCl, 4.7 mM KCl, 1.2 mM MgSO_4_, 1.2 mM KH_2_PO_4_, 2.5 mM CaCl_2_, and 10 mM glucose, pH 7.4). Hearts were cannulated through the aorta and perfused in a Langendorff mode at a constant perfusion pressure of 70 mm Hg at 37 °C with KHB gassed with 95% O_2_–5% CO_2_. Hearts were stabilized for 15 min prior to any interventions. Hearts from the animals assigned to the control group underwent only the stabilization process, while hearts from the I/R groups (I//R and I/R-camphene) were subjected to 30 min of global ischemia by clamping the aortic inflow, followed by either 40 min reperfusion for molecular analysis, or 120 min reperfusion for infarct size determination [11,12].

### 2.3. Infarct Size (IS) Determination

Infarct size was determined using the 2,3,5-triphenyltetrazolium chloride (TTC) method. At the end of reperfusion, hearts were placed at −20 °C for 30 min, cut transversely into 6 slices of 2 mm thickness, and incubated with 1% TTC in 0.1 M phosphate buffer, pH 7.4, for 30 min at 37 °C. Hearts were preserved overnight in PBS buffer (137 mM NaCl, 2.7 mM KCl, 10 mM Na_2_HPO_4_, 1.8 mM KH_2_PO_4_) containing 4% formaldehyde. The infarct size (IS) area was delineated and determined by a computerized planimetric method [28]. The IS was expressed as a percentage of the area at risk, which in the model of global ischemia represents the whole area of the left ventricle.

### 2.4. LDH Determination

Perfusate during the first 20 min of reperfusion of the heart was collected and was used to measure the activity of the enzyme Lactate Dehydrogenase (LDH) that was released from cells with damaged cell membranes [29]. The measurement of the enzyme activity was based on the conversion of NADH at 340 nm (extinction coefficient ε = 6220 M^−1^cm^−1^). The final concentration of the reagents in the reaction mixture was 0.2 M Tris-HCl pH 7.3, 1 mM sodium pyruvate, 0.132 mM nicotinamide adenine dinucleotide hydrogen (NADH), and an appropriate amount of the sample. The rate of conversion of NADH to NAD+ per min, which is proportional to the activity of LDH, was recorded for 5 min. LDH enzyme activity was expressed in U/gr of heart tissue weight.

### 2.5. Determination of Protein Carbonyls

Protein carbonylation was determined by 2,4-dinitrophenyl (DNP) hydrazine (2,4-DNPH) derivatization [30]. Briefly, frozen tissue samples were lysed with 50 mM sodium phosphate buffer pH 6.7 and centrifuged (11,000× *g*, 10 min, 4 °C). Part of the supernatant was kept for determination of total protein concentration. Samples were reacted with 10 mM of 2,4-DNPH in 2 M HCl for 1 h, deproteinized with 20% TCA, and pellets were redissolved in 6 M guanidine hydrochloride. The absorbance was measured at 360 nm and the carbonyl content was calculated using the molar absorption coefficient of 22,000 M^−1^cm^−1^ relative to the protein concentration. Values were expressed as nmol/mg of protein.

### 2.6. Glutathione Determination

GSH levels were estimated using the GSH/GSSG recycling method [31]. Briefly, heart samples were lysed with sulfosalicylic acid 0.6%, Triton-X 0.1%, 0.1 M potassium phosphate buffer, 5 mM EDTA, pH 7.5, and centrifuged (8000× *g*, 10 min, 4 °C). Total glutathione was measured using GSH reductase–DTNB recycling assay and the rate of color developed was measured at 412 nm. GSSG was measured by incubating a portion of the lysate with 2-vinyl pyridine for 1 h at room temperature prior to the recycling assay. Both GSH and GSSG concentrations were calculated using standard curves and expressed in nmol/mg of total protein. GSH was determined by subtracting the GSSG content from the total glutathione content.

### 2.7. Measurement of Citrate Synthase (CS) Activity

For the determination of CS activity, tissue samples were homogenized in 100 mM imidazole, 2 mM EDTA, and 4 mM MgCl_2_, pH 7.6. The citrate synthase assay monitors the rate of production of coenzyme A (CoA.SH) by measuring free sulfhydryl groups using the thiol reagent 5,5′-dithio-bis-(2-nitobenzoic acid) (DTNB). The enzymatic activity was determined spectrophotometrically at 412 nm [32].

### 2.8. Determination of Antioxidant Enzymes Activity

Heart samples were homogenized (1:10 *w*/*v*) in 50 mM sodium phosphate, pH 7.4, sonicated for 3 × 15 s, and centrifuged (11,000× *g*, 10 min, 4 °C). The supernatant was kept on ice for the measurement of enzymatic activities.

#### 2.8.1. Catalase (CAT) Activity Assay

The determination of catalase activity was based on H_2_O_2_ conversion, which was monitored spectrophotometrically at 240 nm (extinction coefficient ε = 0.03941 mM^−1^cm^−1^) [33]. The assay mixture contained 50 mM sodium phosphate buffer, pH 7.4, 30 mM H_2_O_2_, and tissue extract. The rate of the reaction per minute was recorded for 5 min, and the enzyme activity was expressed in U/mg of protein.

#### 2.8.2. Superoxide Dismutase (Mn-SOD) Activity Assay

Mn-SOD activity was determined by the rate of nitroblue tetrazolium (NBT) reduction at 550 nm in a reaction medium containing 50 mM phosphate buffer, pH 8.0, 0.1 mM EDTA, 1 mM potassium cyanide, 0.056 mM NBT, 0.1 mM xanthine, and 8 mU xanthine oxidase [34]. A standard curve with known amounts of SOD was used to calculate enzymatic activity from the % inhibition of NBT reduction in the samples. Enzyme activity was expressed as U/µg protein.

#### 2.8.3. Glutathione Peroxidase (GPx) Activity Assay

The determination of glutathione peroxidase (GPx) activity was based on the reduction in cumene hydroperoxide coupled with the reduction in GSSG by glutathione reductase. The assay mixture contained 50 mM potassium phosphate, pH 7.0, 0.5 mM EDTA, 1 mM GSH, 0.1 Unit of GR, 0.15 mM NADPH, 0.2 mM of cumene hydroperoxide, and tissue extract. The rate of conversion of NADPH to NADP+ was monitored spectrophotometrically at 340 nm (extinction coefficient ε = 6200 M^−1^cm^−1^). The activity of the enzyme was expressed as U/mg of protein.

#### 2.8.4. Glutathione Reductase (GR) Activity Assay

Glutathione reductase was assayed in a mixture containing 50 mM sodium phosphate buffer, pH 7.4, 2 mM EDTA, 1 mM GSSG, 0.15 mM NADPH, and tissue extract. The rate of conversion of NADPH to NADP+ was monitored spectrophotometrically at 340 nm (extinction coefficient ε = 6200 M^−1^cm^−1^). GR activity was expressed as mU/mg of protein [31].

### 2.9. RNA Preparation and Quantitative Real-Time PCR (qPCR)

Total RNA was extracted using TRIzol reagent (Invitrogen, Waltham, MA, USA), as per the manufacturer’s instructions. After extraction, the samples were resuspended in 0.1% (*v*/*v*) diethylpyrocarbonate (DEPC)-treated water, and RNA concentration was determined by absorbance at 260 nm. A total of 1 μg of total RNA was used for cDNA synthesis using the PrimeScript RT Reagent Kit (Takara BIO, Kusatsu, Japan). qPCR analysis was performed using 5 μL KAPA SYBR FAST qPCR Master Mix (Kapa Biosystems, Wilmington, MA, USA), 10 pmol of forward or reverse primer specific for each gene, and 0.05 μg cDNA per reaction. *Actb* expression was used as an endogenous control. The sequences of the primers used are presented in Supplemental Table S1. Relative changes in expression were calculated using the 2 (^−ΔΔCt^) method.

### 2.10. Lipid Peroxidation

The lipid peroxide content in hearts was determined by using the thiobarbituric acid (TBA) reactive material method for the estimation of malondialdehyde content. Hearts were homogenized in TBA reagent containing 15% *w*/*v* TCA, 0.375% *w*/*v* TBA, and 0.25 mol/L HCl. Samples were then boiled for 15 min and cooled on ice for 5 min. Tubes were centrifuged at 2000× *g* for 10 min at 4 °C. The developed color was read at 532 nm. Thiobarbituric acid reactive substances (TBARS) were calculated using a molar extinction coefficient of 1.55 L/mol per cm [35]. The most abundant substance among TBARS is malondialdehyde, (MDA), which is the final product of polyunsaturated fatty acids peroxidation and is recognized as a marker of oxidative stress in humans.

### 2.11. Statistical Analysis

The statistical processing of the results was performed using Prism 9, software, Graph Pad (San Diego, CA, USA). All data were expressed as mean ± standard error (S.E.M) and n refers to individual measurements. To examine statistically significant differences, an analysis of variance (ANOVA) was performed. Tukey’s test was used to compare samples between groups. A difference was considered statistically significant when *p* < 0.05.

## 3. Results

### 3.1. Camphene Pretreatment Reduces Infarct Size and Ameliorates Myocardial I/R Injury

**Figure 1:**
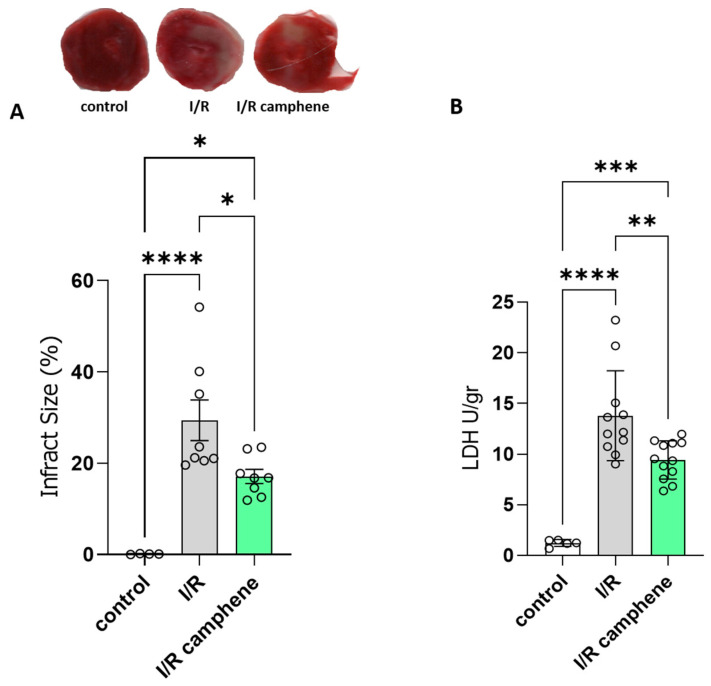
Camphene reduces infarct size in isolated hearts and LDH release following myocardial I/R. (**A**). Representative images of cardiac slices stained with TΤC for each experimental group (**upper panel**) and measured using imaging software (**lower panel**). Infarct size (IS) is expressed as % of risk area (LV), *n* = 4 in control group and *n* = 8 in I/R groups (**B**). LDH release from the perfused heart. Data are presented as U/gr of heart wet wt released in the first 20 min of reperfusion. *n* = 5 in control group, *n* = 11 in I/R group and *n* = 12 in I/R-camphene group. * *p* < 0.05, ** *p* < 0.01, *** *p* < 0.001, **** *p* < 0.0001; one-way ANOVA followed by Tukey’s multiple comparisons test.

To explore the protective role of camphene in response to I/R injury, rat hearts were subjected to ex vivo I/R. Administration of camphene resulted in a significant reduction in infarct size compared with hearts isolated from animals that did not receive any treatment, specifically 29.45% ± 4.44 in I/R hearts and 17.14% ± 1.53 in I/R-camphene (Figure 1A). This result was also accompanied by a decreased release of LDH in the effluent of hearts from animals pretreated with camphene compared with the hearts from non-treated animals, showing that the induction of cell death due to I/R in rat hearts is reduced in the presence of camphene pretreatment (Figure 1B). Specifically, LDH activity was 13.81 ± 1.33 U/gr in the effluent from I/R hearts, while it was 9.44 ± 0.54 U/gr in the effluent of I/R hearts from animals pretreated with camphene.

### 3.2. Camphene Attenuates I/R-Induced Oxidative Stress and Increases Mitochondrial Content

**Figure 2:**
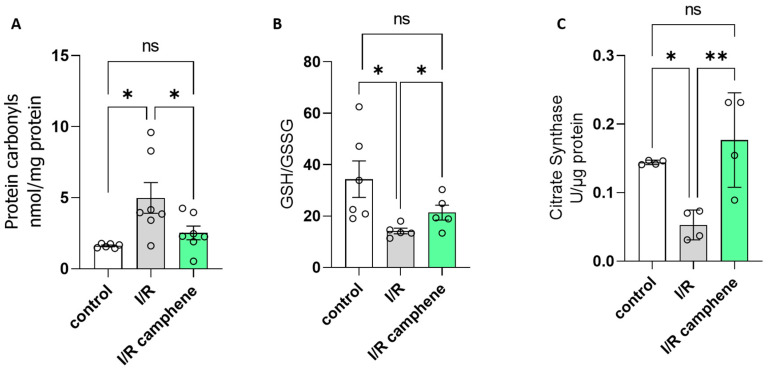
Camphene attenuates oxidative stress and increases mitochondrial content following myocardial I/R (30 min ischemia followed by 40 min reperfusion). (**A**). Protein carbonyls expressed as nmol/mg of total protein. *n* = 6 in control group, *n* = 7 in I/R and I/R-camphene groups. (**B**). The effect of camphene on GSH/GSSG ratio. *n* = 6 in control group, *n* = 5 in I/R and I/R-camphene groups. (**C**). CS activity *n* = 4. * *p* < 0.05, ** *p* < 0.01, ns, non-significant; one-way ANOVA followed by Tukey’s multiple comparisons test.

It is well established that oxidative stress is a major contributing factor in I/R injury. In order to assess the effect of camphene on the level of oxidative stress following myocardial I/R, protein carbonylation levels, as well as the GSH/GSSG ratio, were evaluated. Carbonylation is a post-translational protein modification due to oxidative stress, while the GSH system is the primary defense mechanism against ROS, with the ratio between reduced GSH and the oxidized form GSSG being used as an index of the redox status of cells [7]. As expected, I/R increased the levels of protein carbonyls as a response to oxidative stress from 1.61 ± 0.57 nmol/mg of protein in control hearts to 4.98 ± 1.07 nmol/mg of protein in I/R hearts (Figure 2A). This effect was attenuated in the hearts of animals treated with camphene prior to I/R. Namely, protein carbonyls were 2.52 ± 0.47 nmol/mg of total protein, which is significantly lower than those determined in hearts from non-treated animals (Figure 2A). The beneficial effect of camphene on I/R-induced oxidative stress was confirmed when the GSH/GSSG ratio was determined. The decreased GSH/GSSG ratio observed in the hearts of non-treated animals following I/R was 14.21 ± 1.09, significantly lower than the ratio in control hearts, which was 34.33 ± 7.1. The GSH/GSSG ratio was partially restored to 21.35 ± 2.89 in the hearts of camphene-treated animals (Figure 2B). Furthermore, to assess the effect of camphene on mitochondrial content, which could affect overall mitochondrial respiratory capacity, CS activity was determined [36]. Camphene increased CS activity in the pretreated I/R hearts. CS activity was 0.144 ± 0.001 in control hearts, 0.052 ± 0.01 in I/R hearts, and 0.176 ± 0.03 U/μg protein in I/R hearts from camphene pretreated animals (Figure 2C).

### 3.3. Camphene Pretreatment Activates Antioxidant Mechanisms in the Heart

**Figure 3:**
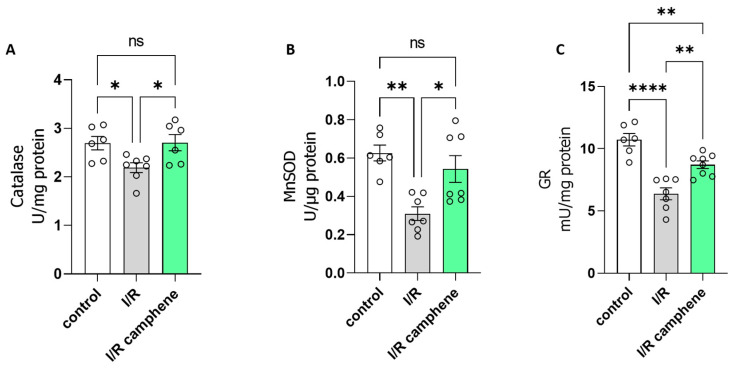
Camphene modulates the activity of antioxidant enzymes after I/R (30 min ischemia followed by 40 min reperfusion). (**A**). Catalase (CAT) activity *n* = 6 in control and I/R-camphene groups, *n* = 7 in I/R group. (**B**). MnSOD activity, *n* = 6 in control group and *n* = 7 in I/R and I/R-camphene groups. (**C**). GR activity, *n* = 6 in control group and *n* = 7 in I/R and I/R-camphene groups. ** *p* < 0.01, * *p* < 0.05, **** *p* < 0.0001, ns, non-significant; one-way ANOVA followed by Tukey’s multiple comparisons test.

To identify the mechanisms underlying the protective role of camphene against oxidative stress induced by I/R, the activity of enzymes related to cellular antioxidant defense was evaluated. The results point to an activation of antioxidant intracellular mechanisms in the presence of camphene. Specifically, the enzymatic activity of catalase (CAT), the enzyme that catalyzes the conversion of H_2_O_2_ into H_2_O and O_2_ in cells, was decreased in I/R while this effect was attenuated in the presence of camphene (Figure 3A). Furthermore, the activity of Mn-SOD, the enzyme that catalyzes the conversion of superoxide radicals to H_2_O_2_ and H_2_O, was reduced by I/R in non-treated animals compared with control hearts, while this downregulation was prevented in hearts from animals treated with camphene prior to the I/R. Specifically, Mn-SOD activity was 0.62 ± 0.04 U/μg in control hearts, 0.32 ± 0.03 U/μg in I/R hearts, and 0.48 ± 0.05 U/μg in I/R-camphene hearts (Figure 3B). Glutathione reductase (GR) is localized in the cytoplasm of cells and catalyzes the conversion of the oxidized form of glutathione (GSSG) to its reduced form (GSH), using the coenzyme NADPH as an electron donor. When GR activity was assessed, it was found to be decreased in I/R hearts compared with control hearts. However, camphene pretreatment ameliorated the I/R observed effect, namely from 6.38 ± 0.48 mU/mg in I/R hearts to 8.71 ± 0.3 mU/mg in I/R-camphene hearts (Figure 3C).

### 3.4. Camphene Pretreatment Decreases Nrf2, HO-1, SOD, and UCP3 mRNA

**Figure 4:**
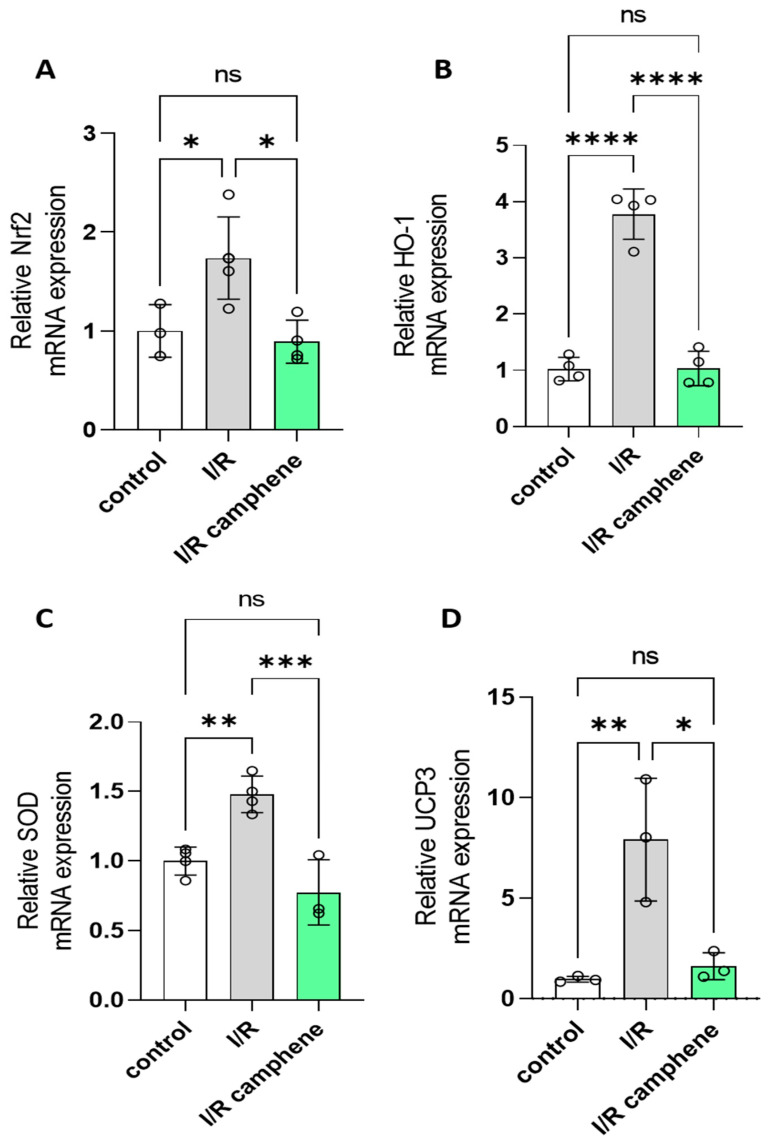
Camphene attenuates the I/R-induced upregulation of Nrf2, HO-1, SOD, and UCP3 mRNA levels. (**A**). Nrf 2 expression levels, *n* = 4. (**B**). HO-1 expression levels, *n* = 4. (**C**). SOD expression levels *n* = 4 (**D**). UCP3 expression levels * *p* < 0.05, ** *p* < 0.01, *** *p* < 0.001, **** *p* < 0.0001, ns, non-significant; one-way ANOVA followed by Tukey’s multiple comparisons test.

To further determine the molecular mechanisms leading to increased antioxidant capacity in the presence of camphene, we determined the mRNA expression of Nrf2, which is a major transcription factor that plays a key role in redox homeostasis in cardiomyocytes. Relative Nrf2 RNA levels were increased in I/R hearts (1.737 ± 0.24) as compared with control hearts (1 ± 0.15) while they returned to basal levels after camphene pretreatment (0.891 ± 0.1 in I/R camphene) (Figure 4A). The upregulation of Nrf2 led to the upregulation of antioxidant enzyme genes, such as HO-1 (Figure 4B). Namely, HO-1 expression was 1.022 ± 0.1, 3.779 ± 0.22, and 1.033 ± 0.15 in control, I/R, and I/R camphene groups, respectively. Similar effects were observed as far as relative SOD and UCP-3 RNA levels were concerned (Figure 4C,D). Namely, expression was 1 ± 0.05, 1.479 ± 0.06, and 0.776 ± 0.13, in control, I/R, and I/R camphene hearts, respectively, for SOD, and, as far as UCP3 was concerned, 0.968 ± 0.08, 7.908 ± 1.76, and 1.617 ± 0.38 in control, I/R, and I/R camphene pretreated hearts, respectively.

### 3.5. Camphene Inhibits Ferroptosis Induced by I/R

**Figure 5:**
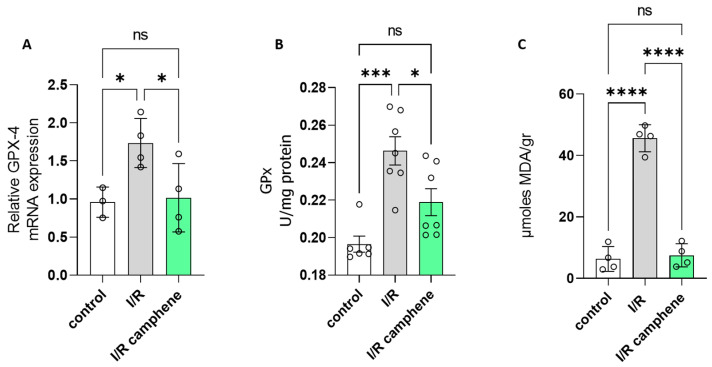
Effect of camphene on markers of ferroptosis. (**A**). Relative mRNA expression of GPx4, n = 4. (**B**). GPx activity, *n* = 6, (**C**). MDA levels *n* = 4. * *p* < 0.05, *** *p* < 0.001, **** *p* < 0.0001, ns, non-significant; one-way ANOVA followed by Tukey’s multiple comparisons test.

Increased expression of glutathione peroxidase (GPx4), the enzyme catalyzing the conversion of hydroperoxides to their corresponding alcohols using GSH as a reducing agent, together with increased lipid peroxides have been associated with ferroptosis-induced cell death [37]. GPx4 mRNA was elevated in the hearts that underwent I/R compared to control hearts, while animal pretreatment with camphene led to attenuation of the I/R observed effect (Figure 5A). Specifically, the relative GPx4 mRNA levels were 0.958 ± 0.11, 1.735 ± 0.16, and 1.016 ± 0.22 in control, I/R, and I/R-camphene hearts, respectively. Accordingly, GPx activity followed the same pattern and was 0.19 ± 0.004 U/mg, 0.24 ± 0.007 U/mg, and 0.21 ± 0.007 in control, I/R, and I/R-camphene hearts, respectively (Figure 5B). Furthermore, lipid peroxidation levels, assessed by the measurement of MDA content were elevated in I/R hearts, with this effect being reduced by camphene pretreatment (Figure 5C). Specifically, MDA levels were 6.32 ± 2.03, 45.63 ± 2.19, and 7.49 ± 1.88 μmoles MDA/gr in control, I/R, and I/R camphene, respectively.

## 4. Discussion

Timely and effective reperfusion of the ischemic myocardium is the only available treatment of choice to date. However, instead of providing salvation, reperfusion can paradoxically intensify injury, known as I/R injury, inducing cardiac arrhythmias, enhancing metabolic defects, and producing structural damage to cardiomyocytes in the heart [38,39]. Several mechanisms have been shown to contribute to I/R, namely the development of oxidative stress and the occurrence of intracellular Ca^2+^ overload, as well as myocardial inflammation and alterations in cardiac metabolism [39,40]. However, it is becoming apparent that oxidative stress is the most critical pathogenetic factor; the production of a large number of ROS during I/R directly or indirectly affects cardiac function, causes cardiomyocyte dysfunction, and promotes cell damage [41,42]. In the present work, we demonstrated that camphene, as a natural product, showed a cardioprotective role in response to myocardial I/R injury, as evidenced by the limitation of infarct size and amelioration of cell damage in the hearts of animals pretreated with camphene. Camphene has a therapeutic effect through the modulation of antioxidant defense mechanisms and, consequently, attenuation of oxidative stress leading to improved overall mitochondrial metabolic activity and attenuation of ferroptotic cell death.

Under physiological conditions, a critical balance exists between ROS production and the endogenous antioxidant system maintaining redox homeostasis. However, under pathological stimuli, including I/R, the excess production of ROS and/or the inability of the antioxidant defense mechanisms to counteract them lead to the opening of the mitochondrial permeability transition pore, lipid peroxidation, and DNA damage, thereby causing cellular dysfunction and death [3,40]. Thus, strategies that prevent or alleviate oxidative stress response are being developed for the treatment of myocardial I/R damage and several studies have reported beneficial effects of antioxidants rendering resistance to the heart against I/R injury. In particular, finding safe and effective drugs derived from natural products is an active research line in this direction. Accumulating evidence suggests that the active components of various medicinal plants, such as flavonoids and phenolics, can protect myocardial cells by regulating oxidative stress and have a good therapeutic effect on I/R injury [14,43,44]. Our study demonstrated that augmented LDH activity and the increased myocardial infarct size elicited by I/R damage were significantly mitigated (Figure 1) in the rats treated with camphene, indicating that camphene attenuated the I/R-induced myocardial injury.

A range of endogenous antioxidants, primarily enzymatic antioxidants (catalase -CAT, superoxide dismutase-SOD, glutathione peroxidase-GPx, glutathione reductase-GR, thioredoxin reductase-TrxR, etc.) are present in the cells to maintain redox homeostasis. Glutathione in its reduced state (GSH) is the most common non-protein thiol in animal cells and it is considered to be one of the most powerful endogenous antioxidant systems in the cardiovascular system due to its key contribution to scavenging overreactive oxygen species (ROS). The scavenging activity of GSH maintains thiol groups of enzymes and other proteins in their reduced state, thus preventing cell membrane lipid peroxidation and limiting cardiomyocyte loss [45,46]. The GSH/GSSG ratio is largely considered a marker of the cellular redox state since it is affected by oxidative stress and in some cases even as a quantitative marker of the severity of the disease. A decrease in the GSH/GSSG levels has been described in animal models of I/R [47] and this was corroborated in our study. However, camphene treatment significantly attenuated this effect, increasing the GSH/GSSG ratio in heart tissues after I/R (Figure 2B). Furthermore, the reduction in oxidative stress in the presence of camphene was confirmed when the levels of protein carbonylation, also extensively used as a marker for cardiac oxidative stress [48], were determined. Treatment of animals with camphene reversed the reduction in myocardial protein carbonyls observed in I/R (Figure 2A). Furthermore, the attenuation of oxidative stress by camphene resulted in an increase in mitochondrial content, as indicated by the increased CS activity in the hearts of camphene-treated animals after I/R as compared with the non-treated ones (Figure 2C), which could have implications on the overall mitochondrial metabolic capacity.

Enzymatic antioxidants such as CAT, SOD, GPx, and GR, which are also crucial components of the cellular defense mechanisms against oxidative stress, are significantly impacted by I/R. However, controversies exist, with some studies demonstrating increased activities during reperfusion as a protective response, while others report decreased activities due to oxidative inactivation [49,50,51]. It seems that the impact of I/R on endogenous antioxidants can vary depending on factors such as the duration and severity of ischemia, the extent of reperfusion injury, and the cellular context [51]. I/R depressed the activities of CAT, Mn-SOD, and GR while camphene treatment reversed this effect (Figure 3A–C). On the other hand, GPx activity was increased in I/R hearts with camphene treatment again attenuating the effect (Figure 5B). Besides the duration of ischemia and/or reperfusion [50,51], GPx activity can differ according to the cellular content of lipid peroxides or even the form of the enzyme, being selenium dependent or independent [52]. Furthermore, I/R injury leads to an increase in ROS production, which subsequently leads to Nrf2 activation to upregulate several target genes of the antioxidant defense [9,10]. Camphene attenuates Nrf2 upregulation induced by I/R, with its target genes HO-1, SOD, and UCP3 following the same pattern (Figure 4). On the other hand, activities of catalase SOD and GR are decreased in I/R while they are significantly increased with camphene pretreatment. Taken together, these results support the notion that camphene acts as a scavenger of intracellular ROS. This is also supported by in vitro studies demonstrating that camphene products, namely camphene thiosemicarbazones, have ROS-scavenging properties [25]. Other natural products such as flavanols have been also shown to have ROS-scavenging capabilities, altering the cellular redox state and, therefore, affecting the expression of antioxidant enzymes [53]. The antioxidant capacity of camphene is also associated with decreased ferroptosis in myocardium, as evidenced by the amelioration of lipid peroxidation and attenuation of GPx4 upregulation. Other studies have also demonstrated that the targeting of ferroptosis could be an alternative to cardiovascular disease treatment [37].

Overall, the results of the present study demonstrate that a single dose of camphene prior to the induction of I/R can attenuate the oxidative stress-induced damage in myocardium. Camphene modulates the activities of endogenous antioxidant systems contributing to the maintenance of cellular redox homeostasis and protecting the cardiomyocytes against ferroptotic cell death. Further research and a better understanding of the mechanism of camphene may provide a novel strategy for preventing myocardial reperfusion injury.

## 5. Conclusions

Camphene effectively protects cardiomyocytes against I/R injury, scavenging ROS activating endogenous antioxidant mechanisms, and maintaining redox balance. Recently, considerable progress has been achieved in treatments designed to reduce oxidative stress during myocardial I/R in vitro and in vivo. In particular, targeted enhancement of the GSH system in the myocardium has been considered a potential therapeutic strategy to prevent myocardial injury [54]. Furthermore, inhibition of ferroptosis can be valuable for myocardium protection against I/R injury [55]. In this respect, camphene may be a promising plant-derived natural compound with therapeutic potential in ischemic heart disease, especially in high-risk populations. However, one of the greatest challenges is to transform the efficacy of preclinical animal research into clinical practice.

### Study Limitations

The present study was designed to determine IS as a robust endpoint of cardioprotection and thus, contractile function was not measured. Although the recovery of ventricular function corresponds generally with the degree of tissue infarction [56], measurement of left-ventricular developed pressure (LVDP), as a secondary endpoint, would be required to further support the cardioprotective effect of camphene. Furthermore, the present work is limited to conclusions based on data from whole heart preparations. Experiments in isolated cardiomyocytes would be required to determine the specific effect of camphene in cardiomyocytes and further support these conclusions.

## Data Availability

Data are contained within the article and Supplementary Materials.

## Supplementary Materials

The following supporting information can be downloaded at: https://www.mdpi.com/article/10.3390/antiox13040405/s1, Table S1: Primers sequences used for qPCR title.

## References

[1] Yellon D.M., Hausenloy D.J. (2007). Myocardial reperfusion injury. N. Engl. J. Med..

[2] Andreadou I., Daiber A., Baxter G.F., Brizzi M.F., Di Lisa F., Kaludercic N., Lazou A., Varga Z.V., Zuurbier C.J., Schulz R. (2021). Influence of cardiometabolic comorbidities on myocardial function, infarction, and cardioprotection: Role of cardiac redox signaling. Free Radic. Biol. Med..

[3] Egea J., Fabregat I., Frapart Y.M., Ghezzi P., Görlach A., Kietzmann T., Kubaichuk K., Knaus U.G., Lopez M.G., Olaso-Gonzalez G. (2017). European contribution to the study of ROS: A summary of the findings and prospects for the future from the COST action BM1203 (EU-ROS). Redox Biol..

[4] Davidson S.M., Adameová A., Barile L., Cabrera-Fuentes H.A., Lazou A., Pagliaro P., Stensløkken K.O., Garcia-Dorado D. (2020). EU-CARDIOPROTECTION COST Action (CA16225). Mitochondrial and mitochondrial-independent pathways of myocardial cell death during ischaemia and reperfusion injury. J. Cell Mol. Med..

[5] Chen Y., Fan H., Wang S., Tang G., Zhai C., Shen L. (2021). Ferroptosis: A Novel Therapeutic Target for Ischemia-Reperfusion Injury. Front. Cell Dev. Biol..

[6] Pizzorno J. (2014). Glutathione!. Integr. Med..

[7] Barteková M., Adameová A., Görbe A., Ferenczyová K., Pecháňová O., Lazou A., Dhalla N.S., Ferdinandy P., Giricz Z. (2021). Natural and synthetic antioxidants targeting cardiac oxidative stress and redox signaling in cardiometabolic diseases. Free Radic. Biol. Med..

[8] Matuz-Mares D., Riveros-Rosas H., Vilchis-Landeros M.M., Vázquez-Meza H. (2021). Glutathione Participation in the Prevention of Cardiovascular Diseases. Antioxidants.

[9] Mata A., Cadenas S. (2021). The Antioxidant Transcription Factor Nrf2 in Cardiac Ischemia–Reperfusion Injury. Int. J. Mol. Sci..

[10] López-Bernardo E., Anedda A., Sánchez-Pérez P., Acosta-Iborra B., Cadenas S. (2015). 4-Hydroxynonenal induces Nrf2-mediated UCP3 upregulation in mouse cardiomyocytes. Free Radic. Biol. Med..

[11] Papatheodorou I., Galatou E., Panagiotidis G.-D., Ravingerová T., Lazou A. (2021). Cardioprotective Effects of PPARβ/δ Activation against Ischemia/Reperfusion Injury in Rat Heart Are Associated with ALDH2 Upregulation, Amelioration of Oxidative Stress and Preservation of Mitochondrial Energy Production. Int. J. Mol. Sci..

[12] Papatheodorou I., Makrecka-Kuka M., Kuka J., Liepinsh E., Dambrova M., Lazou A. (2022). Pharmacological activation of PPARβ/δ preserves mitochondrial respiratory function in ischemia/reperfusion via stimulation of fatty acid oxidation-linked respiration and PGC-1α/NRF-1 signaling. Front. Endocrinol..

[13] Barlaka E., Galatou E., Mellidis K., Ravingerova T., Lazou A. (2016). Role of Pleiotropic Properties of Peroxisome Proliferator-Activated Receptors in the Heart: Focus on the Nonmetabolic Effects in Cardiac Protection. Cardiovasc. Ther..

[14] Tsoumani M., Georgoulis A., Nikolaou P.E., Kostopoulos I.V., Dermintzoglou T., Papatheodorou I., Zoga A., Efentakis P., Konstantinou M., Gikas E. (2021). Acute administration of the olive constituent, oleuropein, combined with ischemic postconditioning increases myocardial protection by modulating oxidative defense. Free Radic. Biol. Med..

[15] Sharmeen J.B., Mahomoodally F.M., Zengin G., Maggi F. (2021). Essential Oils as Natural Sources of Fragrance Compounds for Cosmetics and Cosmeceuticals. Molecules.

[16] de Carvalho H.C., Ieque A.L., Valverde T.L., Baldin V.P., Meneguello J.E., Campanerut-Sá P.A.Z., Vandresen F., Ghi-raldi Lopes L.D., Passos Souza M.R., Santos N.C.S. (2021). Activity of (-)-Camphene Derivatives Against Mycobacterium tuberculosis in Acidic pH. Med. Chem..

[17] de Freitas B.C., Queiroz P.A., Baldin V.P., do Amaral P.H., Rodrigues L.L., Vandresen F., RCaleffi-Ferracioli K., de LScodro R.B., Cardoso R.F., Siqueira V.L. (2020). (-)-Camphene-based derivatives as potential antibacterial agents against *Staphylococcus aureus* and *Enterococcus* spp.. Future Microbiol..

[18] Yamaguchi M.U., Barbosa da Silva A.P., Ueda-Nakamura T., Dias Filho B.P., Conceição da Silva C., Naka-mura C.V. (2009). Effects of a Thiosemicarbazide Camphene Derivative on *Trichophyton mentagrophytes*. Molecules.

[19] Gadotti V.M., Huang S., Zamponi G.W. (2021). The terpenes camphene and alpha-bisabolol inhibit inflammatory and neuropathic pain via Cav3.2 T-type calcium channels. Mol. Brain.

[20] Lin C.T., Chen C.J., Lin T.Y., Tung J.C., Wang S.Y. (2008). Anti-inflammation activity of fruit essential oil from *Cinnamomum inslarimontanum* Hayata. Bioresour. Technol..

[21] Boyd E.M., Sheppard P. (1970). Nutmeg Oil and Camphene as Inhaled Expectorants. Arch. Otolaryngol..

[22] Quintans-Júnior L., Moreira J.C.F., Pasquali MA B., Rabie SM S., Pires A.S., Schröder R., Rabelo T.K., Santos J.P.A., Lima PS S., Cavalcanti S.C.H. (2013). Antinociceptive Activity and Redox Profile of the Monoterpenes (+)-Camphene, p-Cymene, and Geranyl Acetate in Experimental Models. Int. Sch. Res. Not..

[23] Vallianou I., Hadzopoulou-Cladaras M. (2016). Camphene, a plant-derieved monoterpene, exerts its hypolipidemic action by affecting SREBP-1 and MTP expression. PLoS ONE.

[24] Chroni A., Liu T., Gorshkova I., Kan H.Y., Uehara Y., Von Eckardstein A., Zannis V.I. (2003). The central helices of ApoA-I can promote ATP-binding cassette transporter A1 (ABCA1)-mediated lipid efflux. Amino acid residues 220–231 of the wild-type ApoAI are required for lipid efflux in vitro and high density lipoprotein formation in vivo. J. Biol. Chem..

[25] Yang L., Liu H., Xia D., Wang S. (2020). Antioxidant Properties of Camphene-Based Thiosemicarbazones: Experimental and Theoretical Evaluation. Molecules.

[26] Suji B., Jisu K., Byung M. (2020). Camphene Attenuates Skeletal Muscle Atrophy by Regulating Oxidative Stress and Lipid Metabolism in Rats. Nutrients.

[27] Vallianou I., Peroulis N., Pantazis P., Hadzopoulou-Cladaras M. (2011). Camphene, a plant-derived monoterpene, reduces plasma cholesterol and triglycerides in hyperlipidemic rats independently of HMG-CoA reductase activity. PLoS ONE.

[28] Ravingerová T., Matejíková J., Neckár J., Andelová E., Kolár F. (2007). Differential role of PI3K/Akt pathway in the infarct size limitation and antiarrhythmic protection in the rat heart. Mol. Cell Biochem..

[29] Burd J.F., Usategui-Gomez M. (1973). Immunochemical studies on lactate dehydrogenase. Biochim. Biophys. Acta.

[30] Colombo G., Clerici M., Garavaglia M.E., Giustarini D., Rossi R., Milzani A., Dalle-Donne I. (2016). A step-by-step protocol for assaying protein carbonylation in biological samples. J. Chromatogr. B Anal. Technol. Biomed. Life Sci..

[31] Rahman I., Kode A., Biswas S.K. (2006). Assay for quantitative determination of glutathione and glutathione disulfide levels using enzymatic recycling method. Nat. Protoc..

[32] Eigentler A. (2012). Laboratory protocol: Citrate synthase, Mitochondrial marker enzyme. Mitochondr. Physiol. Netw..

[33] Cohen G., Dembiec D., Marcus J. (1970). Measurement of catalase activity in tissue extracts. Anal. Biochem..

[34] Beauchamp C., Fridovich I. (1971). Superoxide dismutase: Improved assays and an assay applicable to acrylamide gels. Anal. Biochem..

[35] Buege J.A., Aust S.T. (1978). Microsomal lipid peroxidation. Methods Enzymol..

[36] Mancilla R., Pava-Mejia D., van Polanen N., de Wit V., Bergman M., Grevendonk L., Jorgensen J., Kornips E., Hoeks J., Hesselink M.K.C. (2023). Invasive and noninvasive markers of human skeletal muscle mitochondrial function. Physiol. Rep..

[37] Chen Z., Yan Y., Qi C., Liu J., Li L., Wang J. (2023). Ferroptosis in cardiovascular diseases: Role and mechanism. Cell Biosci..

[38] Carlberg I., Mannervik B. (1985). Glutathione reductase. Methods Enzymol..

[39] Dhalla N.S., Elmoselhi A.B., Hata T., Makino N. (2000). Status of myocardial antioxidants in ischemia–reperfusion injury. Cardiovasc. Res..

[40] Hausenloy D.J., Yellon D.M. (2013). Myocardial ischemia-reperfusion injury: A neglected therapeutic target. J. Clin. Investig..

[41] Davidson S.M., Ferdinandy P., Andreadou I., Bøtker H.E., Heusch G., Ibáñez B., Ovize M., Schulz R., Yellon D.M., Hausenloy D.J. (2019). Multitarget Strategies to Reduce Myocardial Ischemia/Reperfusion Injury: JACC Review Topic of the Week. J. Am. Coll. Cardiol..

[42] Xiang M., Lu Y., Xin L., Gao J., Shang C., Jiang Z., Lin H., Fang X., Qu Y., Wang Y. (2021). Role of Oxidative Stress in Reperfusion following Myocardial Ischemia and Its Treatments. Oxid. Med. Cell. Longev..

[43] Bradic J., Zivkovic V., Srejovic I., Jakovljevic V., Petkovic A., Turnic T.N., Jeremic J., Jeremic N., Mitrovic S., Sobot T. (2019). Protective Effects of *Galium verum* L. Extract against Cardiac Ischemia/Reperfusion Injury in Spontaneously Hypertensive Rats. Oxid. Med. Cell. Longev.

[44] Draginic N., Milosavljevic I., Andjic M., Jeremic J., Nikolic M., Sretenovic J., Kocovic A., Srejovic I., Zivkovic V., Bolevich S. (2022). Short-Term Administration of Lemon Balm Extract Ameliorates Myocardial Ischemia/Reperfusion Injury: Focus on Oxidative Stress. Pharmaceuticals.

[45] Chang X., Zhang T., Zhang W., Zhao Z., Sun J. (2020). Natural Drugs as a Treatment Strategy for Cardiovascular Disease through the Regulation of Oxidative Stress. Oxid. Med. Cell. Longev..

[46] Tan M., Yin Y., Ma X., Zhang J., Pan W., Tan M., Zhao Y., Yang T., Jiang T., Li H. (2023). Glutathione system enhancement for cardiac protection: Pharmacological options against oxidative stress and ferroptosis. Cell Death Dis..

[47] Daiber A., Andreadou I., Oelze M., Davidson S.M., Hausenloy D.J. (2021). Discovery of new therapeutic redox tar-gets for cardioprotection against ischemia/reperfusion injury and heart failure. Free. Radic. Biol. Med..

[48] Bertero E., Maack C. (2023). Ins and Outs of Glutathione in Cardiac Ischemia/Reperfusion Injury. J. Circ. Res..

[49] Fedorova M., Griesser E., Vemula V., Weber D., Ni Z., Hoffmann R. (2014). Protein and lipid carbonylation in cellular model of nitrosative stress: Mass spectrometry, biochemistry and microscopy study. Free Radic. Biol. Med..

[50] Haramaki N., Stewart D.B., Aggarwal S., Ikeda H., Reznick A.Z., Packer L. (1998). Networking antioxidants in the isolated rat heart are selectively depleted by ischemia-reperfusion. Free Radic. Biol. Med..

[51] Leichtweis S., Ji L.L. (2001). Glutathione deficiency intensifies ischaemia-reperfusion induced cardiac dysfunction and oxidative stress. Acta Physiol. Scand..

[52] Marczin N., El-Habashi N., Hoare G.S., Bundy R.E., Yacoub M. (2003). Antioxidants in myocardial ischemia–reperfusion injury: Therapeutic potential and basic mechanisms. Arch. Biochem. Biophys..

[53] Bouayed J., Bohn T. (2010). Exogenous antioxidants--Double-edged swords in cellular redox state: Health beneficial effects at physiologic doses versus deleterious effects at high doses. Oxid. Med. Cell. Longev..

[54] Aceto A., Mezzetti A., Di Ilio C., Calafiore A.M., De Cesare D., Bosco G., Acciai N., Cappelletti L., Federici G., Cuccurullo F. (1990). Effect of Ischaemia-Reperfusion on Glutathione Peroxidase, Glutathione Reductase and Glutathione Transferase Activities in Human Heart Protected by Hypothermic Cardioplegia, Free Radical. Res. Commun..

[55] Ravingerová T., Kindernay L., Barteková M., Ferko M., Adameová A., Zohdi V., Bernátová I., Ferenczyová K., Lazou A. (2020). The Molecular Mechanisms of Iron Metabolism and Its Role in Cardiac Dysfunction and Cardioprotection. Int. J. Mol. Sci..

[56] Bøtker H.E., Hausenloy D., Andreadou I., Antonucci S., Boengler K., Davidson S.M., Deshwal S., Devaux Y., Di Lisa F., Di Sante M. (2018). Practical guidelines for rigor and reproducibility in preclinical and clinical studies on cardioprotection. Basic. Res. Cardiol..

