# Supplementary material for: Camphene as a Protective Agent in Myocardial Ischemia/Reperfusion Injury
Source: Antioxidants (Basel). 2024 Mar 28;13(4):405. doi: 10.3390/antiox13040405 (PMC11047447; doi:10.3390/antiox13040405)
Supplement: Supplementary file 1 [file antioxidants-13-00405-s001.zip › antioxidants-2804278-supplementary.pdf]

Supplementary material

Table S1. Primers sequences used for the real-time PCR

**Gene**		**sequence**
Actb	For:	GCCCTGAGGCACTCTTCCA
	Rev:	CGGATGTCCACGTCACACTTC
GPx4	For:	ATAAGAACGGCTGCGTGGTGAA
	Rev:	GGCACACACTTGTAGGGCTAGA
Nrf2	For:	CCAGAAGCCACACTGACAGA
	Rev:	GGAGAGGATGCTGCTGAAAG
SOD	For:	GGAGTCCAAGGTTCAGGCT
	Rev:	AGGTAGTAAGCGTGCTCCCA
HO-1	For:	CAGAACCCAGTCTATGCCCC
	Rev:	GTGAGGCCCATACCAGAAGG
UCP3	For:	ACAAAGGATTCATGCCCTCC
	Rev:	GATTCCCGCAGTACCTGGAC
